# Rapid microbiological respiratory point-of-care testing: a qualitative study with primary care clinicians

**DOI:** 10.3399/BJGP.2024.0413

**Published:** 2025-03-11

**Authors:** Rebecca Clarke, Emily Brown, Alastair D Hay, Paul Mark Mitchell, Matthew J Ridd, Liang Zhu, Lucy Yardley

**Affiliations:** School of Psychological Science, University of Bristol, Bristol.; Centre for Academic Primary Care, Population Health Sciences, University of Bristol, Bristol.; Centre for Academic Primary Care, Population Health Sciences, University of Bristol, Bristol.; Health Economics and Health Policy, Population Health Sciences, University of Bristol, Bristol.; Centre for Academic Primary Care, Population Health Sciences, University of Bristol, Bristol.; Health Economics and Health Policy, Population Health Sciences, University of Bristol, Bristol.; School of Psychological Science, University of Bristol, Bristol; School of Psychology, University of Southampton, Southampton.

**Keywords:** antibiotics, diagnostics, point-of-care testing, primary health care, qualitative research, respiratory tract infections

## Abstract

**Background:**

Rapid microbiological point-of-care tests (RM POCTs) present an opportunity to reduce antibiotic exposure and antimicrobial resistance (AMR). So far, there is limited understanding of how RM POCTs may support clinicians in primary care in the UK and how RM POCTs might be integrated into practice.

**Aim:**

To investigate clinicians’ views on how RM POCTs can influence clinical decisions and routine practice, and perspectives on how RM POCTs can impact the clinician–patient relationship.

**Design and setting:**

A qualitative study was undertaken. The study was embedded in a multi-centre, individually randomised controlled efficacy trial, which evaluated the use of a multiplex RM POCT for suspected respiratory tract infections (RTIs) in primary care.

**Method:**

Individual interviews were conducted with 18 clinicians (GPs, *n* = 9; advanced nurse practitioners, *n* = 4; paramedics, *n* = 2; trainee advanced nurse practitioner, *n* = 1; clinical pharmacist, *n* = 1; and emergency care practitioner, *n* = 1). Interviews were audio-recorded, transcribed verbatim, and analysed thematically informed by a realist approach.

**Results:**

RM POCTs can guide prescribing decisions when clinicians experience diagnostic uncertainty and support communication with patients to reinforce prescribing decisions. Consequently, the perceived value of, and use of, RM POCTs varied according to clinicians’ confidence in making prescribing decisions and managing patient expectations and their clinical roles. The costly and time-consuming nature of RM POCTs meant that integration of the tests into routine practice was considered unlikely at present.

**Conclusion:**

The findings from this study highlight the potential benefits and challenges of integrating RM POCTs into routine practice. Clinicians in this study had generally favourable views towards RM POCTs. However, further RM POCT training, complementary strategies, such as communication skills training and patient education, and clear guidance on implementation should be explored to optimise RM POCT feasibility and outcomes across different primary care settings.

## Introduction

Antimicrobial resistance (AMR) poses a significant threat to global health.^1^ In 2019, an estimated 4.95 million deaths were associated with AMR, of which 1.27 million were directly attributable.^2^ The inappropriate prescription of antibiotics has been identified as a key driver of AMR by the World Health Organization.^1^ In the UK, 80% of antibiotics used in health are prescribed through GP practices.^3^ Antibiotic prescription is common for respiratory tract infections (RTIs),^4^ despite evidence to suggest little clinical benefit, as many RTIs are viral or self-limiting.^5^ Factors known to influence prescribing behaviours include perceived pressure from patients and diagnostic uncertainty leading to preventive ‘just-in-case’ prescriptions.^6^ Consequently, there is a need to support clinicians in reducing antibiotic prescriptions safely.^2^

The 2020 Wellcome Trust AMR report^7^ and the O’Neill review on AMR, commissioned by the then UK prime minister,^8^ propose the use of diagnostic tests at the point of patient contact as a solution. Rapid microbiological point-of-care tests (RM POCTs) have the potential to improve diagnostic precision by detecting viruses and bacteria from respiratory tract samples.^9^ Unlike traditional laboratory tests, which take too long to support initial clinical decision making, some RM POCTs can provide results in 15 minutes.^10^ Few studies have explored RM POCTs in primary care in the UK, but promising findings indicate that RM POCTs are acceptable to clinicians, can increase diagnostic certainty, and may improve antimicrobial use.^11^^,^^12^ However, concerns have been raised regarding the limited number of microbes detected, prompting questions about whether clinicians need additional guidance in interpreting results.^11^^,^^13^

There remains a need for greater insight into how RM POCTs can influence clinical decision making and patient management to support successful integration into primary care.^11^^,^^13^^–^^15^ Therefore, this research aimed to explore clinicians’ experiences and views on how RM POCTs can influence clinical decisions and routine practice, and perspectives on how RM POCTs can impact the clinician–patient relationship.

**Table:** How this fits in

Rapid microbiological point-of-care tests (RM POCTs) have the potential to improve diagnostic precision for respiratory tract infections (RTIs) in primary care by detecting viruses and bacteria from respiratory tract samples. So far, little is known about clinicians’ views and experiences of using RM POCTs in primary care. This qualitative study found that RM POCTs were a useful additional tool to support clinical decision making and communication with patients; however, clinicians who were confident in making prescribing decisions and managing patient expectations found RM POCTs less valuable. The time-consuming nature and cost of RM POCTs were considered the biggest barriers to integrating the tests into routine practice.

## Method

### Study setting

This qualitative study was embedded in a multi-centre, individually randomised controlled efficacy trial to evaluate RM POCTs for suspected RTIs in primary care.^16^ Sixteen GP practices in South West England were provided with an RM POCT machine and recruited patients between November 2022 and May 2024. Participating trial clinicians performed nasal and throat swabs on consenting patients who presented to primary care with a suspected respiratory infection when either the patient and/or clinician considered antibiotics were, or might be, necessary. Patients were then randomised to the intervention group (usual care plus RM POCT result) or control group (usual care without RM POCT result). Clinicians were asked to wait for the RM POCT result before treatment decisions were made for patients randomised to the intervention group.

### Sample and recruitment

Convenience sampling was used to recruit clinicians (that is, any clinician usually responsible for managing patients with potential RTIs and who had received RAPID-TEST training) from participating GP practices. All trial clinicians were initially emailed an information sheet regarding the qualitative interviews while the trial was ongoing at their GP practice. These eligibility criteria were modified during qualitative data collection so that only clinicians who had randomised ≥5 patients to the trial and provided them with treatment decisions were contacted about interviews. Clinicians interested in partaking in an interview contacted the research team to arrange an interview. Recruitment continued until data saturation was reached such that no new information was obtained from interviews that would add to the development of new themes.

### Data collection

Individual, semi-structured interviews were conducted remotely or in person between March 2023 and March 2024. Interview topic guides were informed by existing literature and study objectives to explore factors that influenced clinicians’ use of RM POCTs, views on how RM POCTs can influence clinical decisions and routine practice, and perspectives on how RM POCTs can impact the clinician–patient relationship (see Supplementary Information S1). Written, informed consent and demographic data were collected before each interview. Interviews were conducted by the second author (GP and clinical research fellow) and first author (applied health researcher), both of whom are trained and experienced with qualitative research. Interviews lasted an average of 33 minutes and participants received a £40 voucher to thank them for their time.

### Data analysis

Interviews were audio-recorded, transcribed verbatim, and anonymised before transcripts were uploaded into NVivo R1. Thematic analysis was informed using a critical realist perspective,^17^ which was chosen to move beyond exploratory analysis to help understand why intervention resources produce mixed outcomes in different circumstances.^18^ A more explanatory approach is appropriate for complex interventions to construct and refine testable hypotheses for what circumstances, settings, or conditions (contexts) trigger different reactions to intervention resources (mechanisms) and the outcomes from this interaction (outcomes).^18^

First, the first author began a process of data familiarisation and line-by-line, inductive coding to stay close to the data. A subset of transcripts (*n* = 2) was coded by the second author and discrepancies in interpretation were resolved through discussion. Codes were reviewed and refined, and implicit connections between codes led to the formation of themes. Discussion within the multidisciplinary research team was used to explore multiple perspectives and reach a consensus. An iterative approach was taken until a final framework was created and applied to all transcripts.

Following theme generation, the analysis continued under an explanatory approach using abductive reasoning. Abductive reasoning enables researchers to draw on both empirical data and theoretical concepts from existing literature to theorise the underlying drivers that can influence outcomes.^17^ In this instance, abductive reasoning was applied to hypothesise the causal pathways that can lead to positive or adverse outcomes from RM POCT implementation. To achieve this, the first and seventh authors identified and interpreted the contexts in which RM POCTs were implemented, the responses that were triggered (mechanisms), and the resulting outcome. Analysis was repeated across the themes and allowed the development of context–mechanism–outcome (CMO) configurations. This two-step analytical approach aligns with previous research, allowing participants’ narrative experiences to remain foremost while identifying the mechanisms that may drive outcomes.^19^^,^^20^

## Results

The sample comprised 18 clinicians (Table 1). There were 10 female clinicians and eight male clinicians. Most worked in practices located in the least deprived areas, based on the English Index of Multiple Deprivation score.^21^

**Table 1.** Clinician characteristics

**Participant and job title**	**Years qualified in role**	**Gender**	**Practice area deprivation score (range)^a^**
Advanced nurse practitioner 1	8	Female	9
Advanced nurse practitioner 2	15	Female	5
Advanced nurse practitioner 3	18	Male	5
Advanced nurse practitioner 4	2	Female	7
Clinical pharmacist 1	19	Male	9
Emergency care practitioner 1	5	Male	6
GP 1	31	Female	5
GP 2	7	Female	9
GP 3	8	Male	6
GP 4	1	Male	5
GP 5	14	Female	9
GP 6	32	Male	9
GP 7	7	Female	9
GP 8	10	Male	1
GP 9	23	Male	5
Paramedic 1	7	Female	9
Paramedic 2	7	Female	9
Trainee advanced nurse practitioner 1	6	Female	7

a *The English Index of Multiple Deprivation score:^21^ 1 = most deprived, 10 = least deprived.*

Analysis generated the following three themes: clinical decision making; reinforcing diagnosis and supporting the therapeutic relationship; and integrating RM POCTs into routine practice. The CMO configurations generated from these themes and referenced throughout the findings are presented in Boxes 1 and 2.

**Box 1.** Context–mechanism–outcome configurations that facilitate positive outcomes from RM POCT use

**No**	**Context**	**Mechanism**	**Outcome**
**1**	When clinicians are uncertain about appropriate patient management owing to ambiguous clinical markers	… clinicians value the additional insight provided by RM POCTs	… and RM POCTs increase confidence in clinical decision making
**2**	When clinicians fear the adverse outcomes of not prescribing antibiotics owing to uncertainty of symptoms or patient vulnerability	… additional information provided by RM POCTs can reduce clinicians’ concern that patients will deteriorate without antibiotics	… and clinicians feel more confident not prescribing antibiotics when unnecessary
**5**	When clinicians feel uncertain about diagnosis due to clinical inexperience or perceive there to be a clinical responsibility to resolve symptoms	… RM POCTs can help to increase clinicians’ confidence in their clinical suspicions	… and RM POCT results help reduce unnecessary antibiotic prescription and increase confidence in clinical decision making
**7**	When clinicians experience or anticipate patient–clinician conflict owing to patient expectation of antibiotics	… clinicians may perceive RM POCTs to reinforce their diagnosis by demonstrating viral aetiology to patients	… and RM POCT results increase patients’ acceptance of treatment decisions, prevent the therapeutic relationship from breaking down, and reduce the use of delayed antibiotic prescriptions
**9**	When clinicians anticipate conflict or patient reconsulting owing to negative perceptions of their clinical role	… clinicians may perceive RM POCTs to reinforce their diagnosis by demonstrating perceived viral aetiology to patients	… and RM POCT results increase patient trust in the clinical role and avoid the breakdown of the therapeutic relationship
**11**	When clinicians perceive patients to be anxious about symptoms or recovering without antibiotics	… clinicians may perceive RM POCTs to reinforce their diagnosis by demonstrating perceived viral aetiology to patients	… and RM POCT results reduce patient anxiety and increase satisfaction with care and confidence in self-management
**12**	When clinicians perceive patients to have high antibiotic necessity beliefs	… clinicians may perceive RM POCTs to reinforce their diagnosis by demonstrating perceived viral aetiology to patients	… and help educate patients about antibiotic effectivity and reduce the number of patients representing with the same illness and consulting in the future
**15**	When clinicians anticipate that patients will perceive greater advantages to RM POCT use than disadvantages	… clinicians will be motivated to use RM POCTs	… and RM POCTs are used alongside clinical reasoning
**16**	When clinicians are worried about a culture of high antibiotic prescription and AMR, and perceive a clinical responsibility to reduce antibiotic prescribing	… clinicians will be motivated to use RM POCTs	… and RM POCTs are used alongside clinical reasoning
**18**	In a busy clinical context with limited resources (that is, time, finances, and storage space) but adjustments have been made to support RM POCT use	… clinicians will be motivated to use RM POCTs	… and RM POCTs are used alongside clinical reasoning

*AMR = antimicrobial resistance. No = number. RM POCT = rapid microbiological point-of-care test.*

**Box 2.** Context–mechanism–outcome configurations of adverse outcomes from RM POCTs or no changes to clinician behaviour

**No**	**Context**	**Mechanism**	**Outcome**
**3**	When clinicians fear the adverse outcomes of not prescribing antibiotics owing to patient vulnerability	… and perceive RM POCTs to not follow the standard treatment pathway for vulnerable patients	… clinicians will continue to prescribe antibiotics
**4**	When clinicians feel confident about appropriate patient management owing to clear clinical indicators	… and perceive RM POCTs to not provide any additional useful information to support diagnosis or decision making regarding patient prognosis	… clinicians will continue to rely on clinical judgement to support patient management
**6**	When clinicians feel uncertain about diagnosis or unconfident in clinical skills owing to inexperience	… and perceive RM POCT use necessary to support clinical decision making	… clinicians may treat the RM POCT result rather than interpret the result alongside clinical reasoning
**8**	When patients are aware that RM POCTs are available for use in primary care	… clinicians may anticipate or experience patient demand for unnecessary RM POCT use and patient–clinician conflict	… as a result, clinicians may use RM POCTs unnecessarily to prevent the therapeutic relationship from breaking down
**10**	When clinicians anticipate conflict or patient reconsulting owing to negative perceptions of their clinical role	… but perceive RM POCTs to suggest clinicians are unconfident in their clinical judgement and could reinforce distrust	… clinicians will have less motivation to use RM POCTs and will continue to use only clinical judgement to support patient management
**13**	When clinicians perceive patients to have high antibiotic necessity beliefs	… but perceive patients will associate RM POCTs with respiratory symptom management and promote consulting behaviours	… clinicians will continue to use only clinical judgement to support patient management
**14**	When clinicians are confident in their communication skills to resolve challenging patient–clinician conversations	… clinicians perceive RM POCTs to be unnecessary	… and clinicians will continue to use only clinical judgement to support patient management
**17**	In a busy clinical context with limited resources (that is, time, finances, and storage space)	… clinicians will perceive RM POCTs to increase workload stressors, believe the use of RM POCTs should be limited due to the cost, or may be uncertain in their ability to access the RM POCT machine	… as a result, clinicians will have less motivation to use RM POCTs and will continue to use only clinical judgement to support patient management
**19**	When clinicians are uncertain about the cost-effectiveness of RM POCT use	… clinicians may be less motivated to use RM POCTs	… and clinicians will continue to use only clinical judgement to support patient management

*No = number. RM POCT = rapid microbiological point-of-care test.*

### Clinical decision making

Most clinicians shared examples of diagnostic uncertainty and recognised that diagnosis often relies on clinical instinct: *‘There is an element of subjectivity to the decision making’* (advanced nurse practitioner 3). When clinicians were *‘on the fence a little’* (clinical pharmacist 1), they valued additional, objective insight provided by RM POCTs that could help guide diagnosis alongside their clinical reasoning (see CMO1, Box 1).

Additional insight was particularly helpful for situations where diagnostic uncertainty can influence antibiotic prescribing (‘just-in-case’ prescribing). In such situations, viral RM POCT results reduced clinicians’ fear of adverse patient outcomes from bacterial infection and, in turn, reduced unnecessary prescribing:

*‘If I didn’t have it, would I then sit on the more cautious side of the fence in just giving them antibiotics? Potentially, yes.’*

(GP 4)

Clinicians’ fear of adverse outcomes was enhanced in patients perceived to be more vulnerable, such as those with coexisting illnesses and within specific age groups. As a result, some clinicians believed RM POCTs reduced unnecessary antibiotic prescriptions in these groups (see CMO2, Box 1). However, one advanced nurse practitioner demonstrated that opinions varied on whether RM POCTs should be used with vulnerable patients (see CMO3, Box 2):

*‘You just treat them anyway with their amoxicillin and their prednisone, it’s just like a standard treatment pathway; so, I think for those patients, you probably wouldn’t swab them.’*

(Trainee advanced nurse practitioner 1)

Many clinicians emphasised that clinical judgement was still necessary alongside RM POCTs. Clinicians raised concerns that RM POCTs may not identify the cause of symptoms or coexisting illnesses, user error could cause negative test results, or false positive results could occur when commensal bacteria are detected, which are carried by patients but not causative of the current illness. One GP suspected RM POCTs were only *‘99%’* (GP 8) accurate. Thus, it was crucial for clinical reasoning and examinations to remain at the forefront of clinical decision making when establishing the cause of a patient’s illness:

*‘It’s very interesting to know you’ve got a virus, but is that the answer? I don’t think it does help clinical decision making; I suspect not.’*

(GP 1)

Other clinicians expressed a sense of frustration with the ambiguity and interpretation of RM POCT results. While some clinicians suggested that the test would be more useful if RM POCTs also tested for typical bacteria, one clinician emphasised that *‘having an additional module’* (GP 3) would not support clinical decision making about the prognostic significance of bacterial results and whether a patient needs antibiotics:

*‘The question’s actually whether the prognosis is altered by that antibiotic prescription or by that particular finding of that organism there.’*

(GP 3)

Occasionally, when viral test results differed from suspicions of a bacterial infection, a few clinicians reported not prescribing antibiotics. However, when clinicians were confident that there were indicators of a bacterial infection and antibiotics may change the patient’s trajectory, a viral or negative RM POCT result did not alter treatment plans, emphasising their belief in the need for clinical judgement alongside the RM POCT (see CMO4, Box 2):

*‘I’m kind of going on my knowledge of the patient and my experience of similar situations […] I still think that the antibiotic might reduce the severity of their illness. So, I did prescribe antibiotics.’*

(GP 3)

The perceived value of RM POCTs in supporting clinical decision making varied depending on clinical experience and clinicians’ confidence in decision making (see CMO5, Box 1):

*‘That would definitely be really, really, helpful. Especially for somebody like me, who’s newer to this and perhaps not as confident. Whereas you’d probably get some doctors who probably wouldn’t* [use RM POCTs] *because they are very confident in their diagnosing ability.’*

(Trainee advanced nurse practitioner 1)

For some allied healthcare professionals (AHPs), confidence in decision making was not always tied to their clinical skills but to perceptions of clinical responsibility to resolve patients’ symptoms. The use of RM POCTs reassured these clinicians that antibiotics were not always necessary:

*‘It’s that confidence of sort of knowing that it doesn’t matter because they’re … you know, it’s okay, that’s the way you treat it.’*

(Trainee advanced nurse practitioner 1)

The potential for RM POCTs to enhance confidence created a concern that RM POCTs could be relied on for clinical decision making. Clinicians worried that less experienced, less confident clinicians may use RM POCTs as *‘an emotional crutch’* (GP 3) and rely solely on RM POCT results rather than interpret RM POCTs alongside clinical judgement (see CMO6, Box 2).

### Reinforcing diagnosis and supporting the therapeutic relationship

Most clinicians stressed that the objective nature of RM POCTs helped reinforce diagnosis and resolve disagreements when patients were adamant that they needed antibiotics: *‘The benefit is it backs up what we’re saying’* (advanced nurse practitioner 2). Discussing treatment alongside RM POCT results helped to *‘get people on board’* (GP 5) with treatment decisions and reduced the risk of the therapeutic relationship breaking down: *‘The idea of having a kind of third-party test that will arbitrate is sort of quite helpful’* (GP 1). Consequently, RM POCTs enabled a reduction in antibiotic prescriptions often issued when navigating challenging conversations (see CMO7, Box 1):

*‘It’s when I’m saying, “You don’t need antibiotics”, the patient is sitting there saying, “I need antibiotics.” Then maybe now you’d say, “Okay, a delayed prescription, take them in two or three days if you’re not getting any better.” Then you could say actually, “It says you’ve got a viral illness, we’re not going to follow that plan.”’*

(GP 4)

Nevertheless, it was recognised that the availability of RM POCTs may create further patient–clinician conflict in consultations through patient pressure to have unnecessary RM POCTs. Two clinicians proposed that RM POCTs may be used to reduce or avoid this conflict that could stem from patient demand (see CMO8, Box 2).

Evidence to support clinical decisions was thought to be particularly beneficial when doubt and re-consulting occurred owing to patients’ preconceived notions about some clinicians’ ability to diagnose correctly because of their clinical role:

*‘Particularly the patients who are seeing say a paramedic for a first time, there might not necessarily be that kind of relationship of trust. So where are differing opinions absolutely I think an objective measure would be really helpful.’*

(GP 2)

GPs and AHPs believed concordance between RM POCT results and clinicians’ judgement would increase patient trust and improve perceptions about the credibility of certain clinical roles (see CMO9, Box 1). Nevertheless, two advanced nurse practitioners worried that introducing an RM POCT suggested that the clinician does not trust their clinical judgement and could create further patient distrust in the future (see CMO10, Box 2).

Clinicians perceived RM POCTs to provide reassurance to patients with health anxiety by enabling a more objective diagnosis and illness prognosis. Moreover, further investigation of symptoms enhanced patients’ satisfaction with care and validated their experiences of illness (see CMO11, Box 1):

*‘Just having a name for it, they kind of go “ah, okay, that’s what I’ve got” rather than the mind wandering into all kinds of “what have I got?”’*

(Clinical pharmacist 1)

*‘Being able to say, “Yeah, I believe you and actually here’s the data as well to show in addition that I believe you.”’*

(GP 5)

Some clinicians believed RM POCTs had a role in primary care to support certain patient groups, such as vulnerable patients, who had low self-efficacy beliefs and often re-presented and required further reassurance in their ability to self-manage their illness and recover without antibiotics. However, one GP suggested that negative test results may raise patient anxiety and some patients may need further reassurance from clinicians.

The educational potential of RM POCTs was also considered. Some clinicians believed objective measures, such as RM POCTs, strengthened communication about antibiotic effectiveness and taught patients which symptoms were associated with viral or bacterial illnesses (see CMO12, Box 1):

*‘They’ve gone, “Oh, I didn’t realise I had the flu, I thought that the flu would exhibit symptoms A, B, and C and actually I was presenting with symptoms X, Y, and Z. Now I know what I’ve got.”’*

(Advanced nurse practitioner 3)

The belief that this experience could reduce re-presenting or future consulting and lessen antibiotic prescription was challenged by one clinician who explained two patients who had tested positive for a virus *‘both then ended up on the doctor’s list a couple of days later’* (paramedic 1).

Other clinicians expressed concern that, instead of RM POCTs reinforcing educational messages and enhancing self-management beliefs, patients may learn to associate RM POCTs with managing respiratory symptoms. As a result, RM POCTs could increase consulting behaviours and antibiotic prescriptions when some patients may usually self-manage without visiting primary care (see CMO13, Box 2).

Moreover, some clinicians believed that RM POCTs should not be necessary or relied on to resolve conflict, reassure patients, or educate as confident communication should be enough (see CMO14, Box 2): *‘If we’re confident usually the patient will have confidence with us’* (clinical pharmacist 1). However, it was believed that confidently communicating to reinforce clinical decision making and address medication beliefs was more challenging for less experienced clinicians, particularly without an established rapport with a patient.

### Integrating RM POCTs into routine practice

Clinicians considered it optimal timing to introduce RM POCTs for RTIs to primary care and perceived most patients to respond favourably following the COVID-19 outbreak. However, it was recognised that there would still be some patients who are less accepting owing to discomfort experienced or worry that rapid-test results required isolation (see CMO15, Box 1).

Some clinicians shared their worry about AMR and its impact on patients and the NHS. Therefore, clinicians had a clinical responsibility to use available tools such as RM POCTs to try to reduce antibiotic prescribing and optimise patient outcomes (see CMO16, Box 1):

*‘A bunch of us need to be thinking, let’s try and reduce the fuel that’s going on the fire so that we’re not just dishing out antibiotics left, right and centre.’*

(Advanced nurse practitioner 3)

Nevertheless, clinicians questioned how RM POCTs would fit into routine practice after the trial owing to the availability of resources (see CMO17, Box 2). Concerns arose for all clinicians around the time to conduct and produce RM POCT results, and the additional appointment time needed to deliver results. One clinician suggested time-consuming RM POCTs may unintentionally increase workload stressors and prescribing:

*‘So, you might in fact find that actually there is a paradoxical increase in the prescribing of antibiotics because the workload pressure for all other conditions that are not doing the test goes up.’*

(GP 3)

Consequently, time was the biggest barrier to RM POCT use.

Clinicians further considered the financial implications of using RM POCTs. In particular, GPs stressed that RM POCT use would be limited or better placed in secondary care or pharmacies if the cost had to come from practice budgets: *‘We can’t afford it so how are we going to manage that, you know?’* (GP 1). One GP suggested that costs needed to be reimbursed to incentivise use: *‘Could we prescribe the test or get funding back to actually be able to use the test?’* (GP 1). In contrast, other clinicians who were not responsible for practice finances believed the benefits of RM POCTs would outweigh the cost:

*‘Sometimes you’ve got to take the hit financially, haven’t you, to change practice and change patient views on things.’*

(Advanced nurse practitioner 1)

The size and noise of the RM POCT machine also created challenges. Placement within clinic rooms disrupted consultations owing to the sound produced, and access to the machine was limited while clinic rooms were in use. However, not all practices had space to store the machine elsewhere.

Despite previous emphasis on the necessity of clinical judgement alongside RM POCTs, some clinicians discussed using RM POCTs as a way to alleviate workload burdens by triaging patients. A few clinicians suggested that, if RM POCTs were conducted before consultations, viral results and safety netting may *‘take away the whole need even for consultation’* (GP 4). Alternatively, RM POCTs could support remote consultations if the tests were conducted before appointments. Nevertheless, it was acknowledged that this could risk patient safety.

Clinicians made other recommended changes to reduce burdens associated with RM POCTs. Such recommendations included training less costly staff, such as urgent care clinicians, healthcare assistants, and administrators, to perform and process RM POCTs, and processing multiple RM POCTs together:

*‘All the samples could be put to one side and run all in one go, one after another, over an admin period, where someone from the admin team, or the healthcare assistant team, could just run those.’*

(Clinical pharmacist 1)

Allocating additional appointments for patients who may need RM POCTs would reduce the time burden placed on clinicians (see CMO18, Box 1). Nevertheless, some clinicians doubted whether reducing appointment availability was justifiable. One GP recognised smaller practices have fewer resources; therefore, supporting RM POCT use would be more challenging.

Consequently, some clinicians believed the cost-effectiveness of RM POCTs in primary care needed to be assessed. Most AHPs were optimistic about the cost-effectiveness of RM POCTs, while GPs questioned *‘whether the cost of the test in both staff time and financial cost could be justified’* (GP 1). Therefore, evidence was needed to see whether RM POCTs optimised patient care and reduced re-presenting and prescribing rates before implementation (see CMO19, Box 2).

## Discussion

### Summary

Our findings provide insight into how RM POCTs can help guide prescribing decisions when clinicians experience diagnostic uncertainty and support communication with patients by negotiating differences in treatment expectations. The perceived value of RM POCTs was related to clinicians’ level of confidence in making prescribing decisions and convincing patients that these were correct; clinicians with less experience were, therefore, more likely to value them. While considered a useful additional tool alongside clinical judgement, the time-consuming nature and cost were considered barriers to the widespread adoption of RM POCTs at the present time, and some concerns were expressed about the risk of overreliance on RM POCTs by less experienced clinicians.

### Strengths and limitations

This study benefited from a varied sample of clinical roles. While some clinical roles are underrepresented, the breadth of clinical roles reflects the range of clinicians in primary care in the UK. Nevertheless, the clinicians in this study mostly worked in practices located in the least deprived areas, which may have influenced clinicians’ experiences with patients and views of point-of-care tests.^22^

Two different researchers interviewed the participating clinicians, and it is possible that the interview guides were interacted with differently. Nevertheless, an insider and outsider interviewer perspective (clinician and applied health researcher) likely helped capture a greater variety and depth of data. It is also worth considering that clinicians’ views may differ from observable data and may not reflect patients’ perspectives of RM POCT use. Further quantitative analysis and the qualitative findings exploring patients’ experiences with RM POCTs in the RAPID-TEST trial will be reported separately.^16^ Furthermore, this study follows guidance from the Medical Research Council^23^ and critical realist principles to consider the context and mechanisms that can generate different outcomes in an intervention. While we explicitly focus on the immediate context of the clinician–patient dyad, primary care, and wider influences raised by participants, we recognise that further contexts influence prescribing behaviours that have not been accounted for.

### Comparison with existing literature

Similar to previous research on C-reactive protein point-of-care tests (CRP POCTs),^24^^–^^26^ our findings indicate that additional information from RM POCTs can enhance clinicians’ confidence to withhold antibiotics and alleviate concern about adverse clinical outcomes. This finding aligns with insights from social cognitive theory,^27^ which underpinned the theory of change that guided this intervention. This theory of change predicted that increasing clinicians’ self-efficacy and positive outcome expectations could reduce their antibiotic prescribing when the point-of-care test indicated that a virus was a likely cause of symptoms. Addressing these mechanisms may be particularly useful for less experienced, more risk-averse clinicians or AHPs whose prescribing behaviours are influenced by perceptions about their clinical role.^28^ However, worry about conducting tests correctly and ambiguity in interpretation may sustain clinicians’ concerns about adverse outcomes.^29^ Additionally, reliance on RM POCTs to address low self-efficacy beliefs poses the risk of inappropriate use and reduced clinical reasoning, which was a concern raised by RAPID-TEST patients (Clarke R, manuscript submitted for publication, 2025).

Clinicians in this study perceived using RM POCTs as valuable for managing patient expectations for antibiotics and enhancing patient satisfaction. These findings complement previous evidence that RM POCTs and CRP POCTs can help justify prescribing decisions, provide reassurance to patients, and improve the professional credibility of AHPs.^11^^,^^26^^,^^30^^,^^31^ However, clinicians in the present study extend this understanding by highlighting RM POCTs could undermine AHPs’ clinical skills and exacerbate distrust. Furthermore, previous research has highlighted that additional communication challenges can arise from CRP POCTs, such as patient misunderstanding and dissatisfaction when results do not align with patient experience.^25^^,^^26^^,^^30^ Indeed, in this trial some patients expressed dissatisfaction and doubt in RM POCT results, and these negative views appeared to be related to their illness experience, antibiotic expectations, and concerns about AHP expertise (Clarke R, manuscript submitted for publication, 2025). In an increasingly digitalised environment, where access to health information online can influence patient requests for tests,^32^ clinicians may also have to manage patient expectations of access to testing. Previous trials have suggested that communication skills training and tools alongside CRP POCT use can help to increase clinician confidence in addressing patient expectations.^30^^,^^33^ It is worth noting, however, that some evidence suggests that communication skills training is more efficacious in the long term in reducing antibiotic prescribing than CRP POCTs.^34^

In line with the normalisation process theory,^35^ clinicians also assessed the value of RM POCTs by determining how easily they fit into current working practices and whether the benefits outweighed the costs. Consistent with existing research using CRP POCTs, participants in this study considered the additional resource burden of RM POCTs the primary barrier to routine adoption,^24^^,^^26^^,^^36^ highlighting the need for health economic analysis in this area. These views contrast with some RAPID-TEST trial patients who believed RM POCTs would financially benefit GP practices and reduce the pressure on primary care (Clarke R, manuscript submitted for publication, 2025). While clinicians in this study recommended changes to support RM POCT use, it was acknowledged that the effectiveness of such changes would vary depending on practice size. Clinicians responsible for practice finances perceived RM POCTs as less valuable, highlighting that RM POCTs are unlikely to be adopted until these barriers are addressed.^36^ Reimbursement models, the development of quicker and smaller RM POCTs, and guidance for practices on integrating RM POCTs, without creating additional burdens, are needed.^37^

### Implications for research and practice

Findings indicate that RM POCTs can reduce clinical uncertainty and fear of adverse outcomes, and help manage patient expectations by reinforcing diagnosis. However, it is clear that not all clinicians found RM POCTs valuable. Combining point-of-care tests to manage RTIs with complementary strategies, such as communication skills training and patient education, has previously been recommended to enhance the effectiveness of RM POCTs.^37^ The identification of CMOs in this study elucidates the contexts where complementary strategies may be most beneficial. Future research should explore how to optimise different complementary strategies in a way to minimise the burden placed on clinicians and GP practices. Consideration of ways to safely integrate RM POCTs as a triage tool may be one way to reduce clinical burden.

The value of RM POCTs and how they support clinical decision making was tied to clinical experience and perceptions about clinical identity. Clear national guidance and training on using RM POCTs is essential to ensure the tests are used alongside clinical reasoning and to help reduce clinicians’ concerns about accurate RM POCT interpretation.^37^ This finding also has important implications for the growing range of clinicians managing RTIs in different healthcare contexts across the UK (for example, community pharmacies). Previous calls for bespoke guidance on using point-of-care tests in the community highlight the need for further research to understand the barriers and facilitators to RM POCT implementation across different settings and clinical roles.^29^^,^^38^

## Data

Further data are available from the corresponding author upon reasonable request.
